# Clinical activity of nivolumab in patients with non-clear cell renal cell carcinoma

**DOI:** 10.1186/s40425-018-0319-9

**Published:** 2018-01-29

**Authors:** Vadim S. Koshkin, Pedro C. Barata, Tian Zhang, Daniel J. George, Michael B. Atkins, William J. Kelly, Nicholas J. Vogelzang, Sumanta K. Pal, JoAnn Hsu, Leonard J. Appleman, Moshe C. Ornstein, Timothy Gilligan, Petros Grivas, Jorge A. Garcia, Brian I. Rini

**Affiliations:** 1 Department of Hematology & Medical Oncology, Taussig Cancer Institute, Cleveland Clinic, 9500 Euclid Ave, Desk CA60, Cleveland, OH 44195 USA; 2 Department of Medicine, Division of Medical Oncology, Duke University Medical Center, Durham, NC USA; 3 Georgetown Lombardi Comprehensive Cancer Center, Washington, DC USA; 4 US Oncology Research/Comprehensive Cancer Centers of Nevada, Las Vegas, NV USA; 5 Department of Medical Oncology & Therapeutics Research, City of Hope Comprehensive Cancer Center, Duarte, CA USA; 6 Division of Hematology/Oncology, University of Pittsburgh, Pittsburgh, PA USA; 7 Fred Hutchinson Cancer Research Center, University of Washington, Seattle, WA USA

**Keywords:** Non-clear cell renal cell carcinoma, mRCC, Nivolumab, Immunotherapy, PD1/PDL1 pathway, Checkpoint inhibitor

## Abstract

**Background:**

Nivolumab is approved for patients with metastatic renal cell carcinoma (mRCC) refractory to prior antiangiogenic therapy. The clinical activity of nivolumab in patients with non-clear cell RCC subtypes remains unknown as these patients were excluded from the original nivolumab trials.

**Methods:**

Patients from 6 centers in the United States who received at least one dose of nivolumab for non-clear cell mRCC between 12/2015 and 06/2017 were identified. A retrospective analysis including patient characteristics, objective response rate according to RECIST v1.1 and treatment-related adverse events (TRAEs) was undertaken.

**Results:**

Forty-one patients were identified. Median age was 58 years (33–82), 71% were male, and majority had ECOG PS 0 (40%) or 1 (47%). Histology included 16 papillary, 14 unclassified, 5 chromophobe, 4 collecting duct, 1 Xp11 translocation and 1 MTSCC (mucinous tubular and spindle cell carcinoma). Among 35 patients who were evaluable for best response, 7 (20%) had PR and 10 (29%) had SD. Responses were observed in unclassified, papillary and collecting duct subtypes. In the entire cohort, median follow-up was 8.5 months and median treatment duration was 3.0 months. Median PFS was 3.5 months and median OS was not reached. Among responders, median time to best response was 5.1 months, and median duration of response was not reached as only 2 out of 7 responders had disease progression during follow-up. TRAEs of any grade were noted in 37% and most commonly included fatigue (12%), fever (10%) and rash (10%). Nivolumab treatments were postponed in 34% and discontinued in 15% of patients due to intolerance. No treatment-related deaths were observed.

**Conclusions:**

Nivolumab monotherapy demonstrated objective responses and was well tolerated in a heterogeneous population of patients with non-clear cell mRCC. In the absence of other data in this treatment setting, this study lends support to the use of nivolumab for patients with metastatic non-clear cell renal cell carcinoma.

## Background

Clear cell renal cell carcinoma (ccRCC) represents up to 75–85% of primary kidney malignancies, while other histologies known collectively as non-clear cell renal cell carcinomas (non-ccRCC) account for the remaining 15–25% [1]. Non-ccRCC encompasses a heterogeneous group of tumors including papillary, chromophobe, collecting duct, translocation, medullary and unclassified subtypes [2, 3]. These histologic subtypes have pathologic and molecular features distinct from ccRCC and often display different clinical phenotypes [3]. A large meta-analysis including 49 studies and 1244 patients with non-ccRCC who were treated with anti-angiogenic and targeted agents approved for treatment of ccRCC, demonstrated that non-ccRCC patients had lower ORR, OS, and PFS compared to patients with ccRCC included in these studies [4]. Randomized trials comparing sunitinib to everolimus as first-line treatment in non-ccRCC have shown modest clinical activity of these agents in patients with non-ccRCC, with lower response rates compared to those observed in patients with clear cell histology [5, 6]. In addition to lower response rates, certain non-ccRCC histologies (e.g. unclassified, translocation and collecting duct) have more aggressive biology and worse survival compared to ccRCC [7–10]. Due to the limited prospective evidence for treatment of non-ccRCC, treatment strategies for these patients are often extrapolated from prospective trials in patients with ccRCC [3, 11].

Nivolumab is a humanized monoclonal anti-PD1 antibody whose approval in mRCC was based on the data from CheckMate 025, a randomized phase III trial of nivolumab compared to everolimus in patients with refractory mRCC that demonstrated an overall survival advantage for patients treated with nivolumab [12–14]. Despite its widespread use in patients with refractory mRCC including patients with a non-clear cell histology, the clinical activity of nivolumab in non-ccRCC patients remains unknown as they were not part of the original trial population. A multicenter, retrospective analysis of the efficacy and safety of nivolumab monotherapy in mRCC patients with non-clear cell histology was undertaken to address this evidence gap.

## Methods

A total of 41 patients from 6 institutions in the United States (Cleveland Clinic, Duke, Georgetown, Comprehensive Cancer Centers of Nevada, City of Hope and University of Pittsburgh Medical Center) who were treated with nivolumab between December 2015 and June 2017 were included in this retrospective analysis. Patient data were collected in compliance with the IRB guidelines of each participating institution.

Patient eligibility criteria for this analysis included: histologically confirmed non-clear cell RCC, presence of metastatic disease, at least one dose of nivolumab monotherapy administered and available clinical and imaging data prior to initiation of treatment. To be considered eligible for response assessment patients needed to have at least one scan following initiation of nivolumab treatment or to have had clinical progression following initiation of nivolumab treatment as assessed by the treating physician. The demographic, clinical and treatment data for each patient were obtained from retrospective chart review by investigators at each institution. Patient scans prior to initiation of nivolumab and while on nivolumab treatment were reviewed locally by a participating investigator at each institution. RECISTv1.1 was used to define objective response rate (ORR). Duration of follow-up was defined as the time from the date of first nivolumab dose to the date of last follow up or documented date of death. Progression free survival (PFS) was defined as the time from initiation of treatment to the time of progression or death, while overall survival (OS) was defined as the time from initiation of treatment until the time of death. Duration of nivolumab treatment was defined as the time from the date of first nivolumab dose to the date of last nivolumab dose. Time to best response was defined as the time from the date of first nivolumab dose until the date of initial documentation of best treatment response. Duration of response was defined for patients achieving CR or PR as the time from initial documentation of response until the date of radiographically confirmed PD. Descriptive statistics were used to tabulate patient and treatment characteristics as well as treatment outcomes and rates of treatment-related adverse events (TRAEs).

## Results

### Baseline patient characteristics

A total of 41 patients received at least one dose of nivolumab. The median patient age at the time of nivolumab initiation was 58 (range, 33–82). The majority of patients were male (71%) and had ECOG performance status of 0 or 1. This was a diverse patient population that was 67% Caucasian, 25% African American and 8% Hispanic (race was unknown for one patient).

The most common histology was papillary (16 patients, 39%), followed by unclassified (14 patients, 34%), chromophobe (5 patients, 12%), and collecting duct (4 patients, 10%) (Table 1). One patient had Xp11 translocation carcinoma and another patient had mucinous tubular and spindle cell carcinoma (MTSCC). Sarcomatoid component was noted in 5 patients (12%). The risk group breakdown according to MSKCC criteria for patients who have had prior systemic treatment was 21% favorable, 64% intermediate and 15% poor [15], and according to IMDC criteria was 27% favorable, 62% intermediate and 11% poor [16]. The majority of patients (73%) had a prior nephrectomy, and for most patients the nephrectomy sample was used to establish the histologic diagnosis of non-clear cell RCC. Metastatic disease was present at the time of original diagnosis in slightly more than half (54%) of patients. The most common location of metastases included: retroperitoneal lymph nodes 63%, lung 54%, liver 37%, bone 27%, and mediastinal lymph nodes 27%. The majority of patients had 1–2 prior systemic therapies (82%), although 4 patients (10%) had three or more prior systemic treatments and 3 patients (8%) had no systemic treatment prior to nivolumab. The most common prior systemic treatments included sunitinib (63%), pazopanib (27%), axitinib (10%) and everolimus (10%). None of the patients included in the analysis had prior IL-2 treatment.

**Table 1:** Baseline patient, disease and prior treatment characteristics

Characteristics	*N* = 41
Median Age (Range)	58 (33–82)
Gender	Male: 29 (71%)
	Female: 12 (29%)
ECOG PS	0: 15 (40%)
	1: 18 (47%)
	2: 5 (13%)
	Unknown: 3
Race	Caucasian: 27 (68%)
	African American: 10 (25%)
	Hispanic: 3 (7%)
	Unknown: 1
Histology	Papillary: 16 (39%)
	Unclassified: 14 (34%)
	Chromophobe: 5 (12%)
	Collecting Duct: 4 (10%)
	Translocation: 1 (2%)
	Mucinous Tubular and Spindle Cell Carcinoma (MTSCC): 1 (2%)
Risk Group	MSKCC Criteria [15]
	Favorable: 8 (21%)
	Intermediate: 25 (64%)
	Poor: 6 (15%)
	Unknown: 2
	IMDC Criteria [16]
	Favorable: 10 (27%)
	Intermediate: 23 (62%)
	Poor: 4 (11%)
	Unknown: 4
Location of Metastases (more than 1 possible)	Retroperitoneal LNs: 26 (63%)
	Lung: 22 (54%)
	Liver: 15 (37%)
	Bone: 11 (27%)
	Mediastinal LNs: 11 (27%)
	Supraclavicular LNs: 7 (17%)
	Mesenteric LNs: 5 (12%)
	Pelvic Mass: 4 (10%)
	Adrenal: 4 (10%)
	Omentum: 3 (7%)
	Pancreas, brain, contralateral kidney, soft tissue: 2 each (5%)
	Spleen, diaphragm, gallbladder, iliac LNs: 1 each (2%)
Prior Nephrectomy	29 (73%)
Number of Prior Systemic Therapies	0: 3 (8%)
	1: 25 (62%)
	2: 8 (20%)
	3 or more: 4 (10%)
	Unknown: 1
Prior Therapies (more than 1 possible)	Sunitinib: 26 (63%)
	Pazopanib: 11 (27%)
	Axitinib: 4 (10%)
	Everolimus: 4 (10%)
	Cabozantinib: 3 (7%)
	Gemcitabine/Cisplatin: 3 (7%)
	Carboplatin/Taxol: 1 (2%)
	Sorafenib: 1 (2%)
	Bevacizumab/Everolimus: 1 (2%)
Prior Immunotherapy	Atezolizumab on trial: 1 (2%) Ipi/Nivo trial and J14186 HAR vaccine trial: 1 (2%)

In some categories percentages do not add up to 100% due to rounding

*Abbreviations*: *ECOG PS* Eastern Cooperative Oncology Group Performance Status, *MSKCC* Memorial Sloan Kettering Cancer Center, *IMDC* International Metastatic Renal Cell Carcinoma Database Consortium, *LNs* Lymph Nodes

### Nivolumab response rates and outcomes

**Fig. 1:**
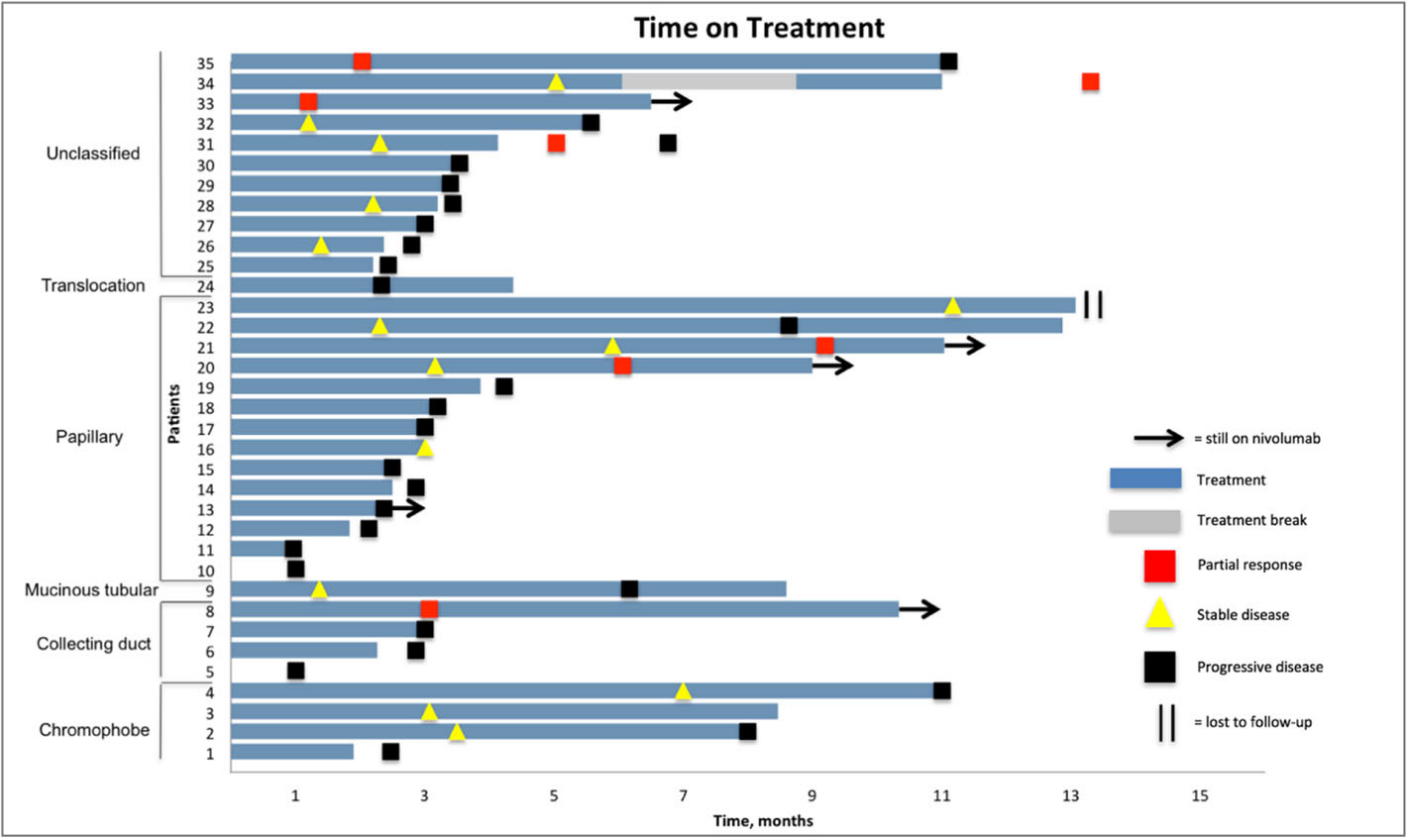
Swimmer’s Plot of Time on Treatment for 35 Evaluable Patients. Patient 5 and patient 10 received a single dose of nivolumab. Patient 31 and patient 34 had documented PR after discontinuation of nivolumab due to intolerance

A total of 35 patients were considered evaluable for treatment response. The objective response rate (ORR) determined by local investigators according to RECIST v1.1 was 20% (7 patients), all PRs. Stable disease (SD) was noted as the best response for 29% (10 patients). The remaining 51% (18 patients) had progressive disease (14 patients with radiographic PD and 4 patients with clinical PD) as the best response. Among the 6 patients who were considered not evaluable, two stopped nivolumab after only one infusion due to treatment intolerance, one patient was lost to follow-up after transferring care to another institution, and another three patients were still on treatment and had not yet had a scan to assess treatment response. Response rates according to RCC subtypes are shown in Table 2. Partial responses were seen in patients with papillary, unclassified and collecting duct histologies, whereas 3 of 4 patients with chromophobe histology had SD with no observed responses. Among five patients whose biopsies had sarcomatoid component, 2 patients had PR, 2 patients had SD and 1 patient had PD as best response. Among patients who had an objective response to treatment, the mean percentage of tumor change was − 38%. Among patients with PR or SD as best response, mean percentage of tumor change was − 20%. Two patients were assessed as having clinical PD (and recorded as having PD) despite having scans at the time of presentation that were consistent with SD based on RECIST v1.1. Another two patients were recorded as having PD after having documented clinical disease progression following just one dose of nivolumab. A total of 4 patients were continued on nivolumab treatment beyond radiographic progression. Two patients had documented radiographic PR after nivolumab was discontinued due to treatment intolerance. A swimmer’s plot of treatment duration for all 35 evaluable patients is shown in Fig. 1.

**Table 2:** Best response to nivolumab (RECIST v 1.1) based on RCC histology

Histology	*N*	PR	SD	PD	Non-evaluable
Papillary	16	2 (14%)	3 (21%)	9 (64%)	2
Unclassified	14	4 (36%)	3 (27%)	4 (36%)	3
Chromophobe	5	0 (0%)	3 (75%)	1 (25%)	1
Collecting Duct	4	1 (25%)	0 (0%)	3 (75%)	0
MTSCC	1	0 (0%)	1 (100%)	0 (0%)	0
Translocation	1	0 (0%)	0 (0%)	1 (100%)	0
All Histologies	41	7 (20%)	10 (29%)	18 (51%)	6

No complete responses (CRs) were observed in this study

For some histologies total percentages do not add up to 100% due to rounding

*Abbreviations*: *RECIST* Response Criteria in Solid Tumors, *PR* Partial Response, *SD* Stable Disease, *PD* Progressive Disease, *MTSCC* Mucinous Tubular and Spindle Cell Carcinoma

In the overall 41 patient cohort, the median duration of nivolumab treatment was 3.0 months (range, 0–13.1 months) and the median number of nivolumab doses received by patients was 7 (range, 1–28). After a median follow-up of 8.5 months (range, 0.6–18.4 months) the median time to best response among patients with PR was 5.1 months (range, 1.2–13.3). The median duration of response among patients with PR was not reached at the time of analysis, however among the 7 patients with PR, 5 had ongoing responses at the time of analysis and 4 patients were still receiving nivolumab. Among the 3 patients who discontinued nivolumab after initial response to treatment, two discontinued treatment due to disease progression and one patient due to treatment intolerance. At the time of analysis, 4 out of 7 responders had response duration of at least 4 months.

A total of 27 patients had disease progression during the follow-up period and 13 patients died (11 after documented disease progression). The median PFS was 3.5 months (95% CI: 1.9–5.0 months). Median OS was not reached, and the overall survival at the ten-month time point from the start of nivolumab treatment was 68% (17/25). The majority of patients who had disease progression on nivolumab treatment were able to receive subsequent systemic treatment (18 of 27 patients, 67%), and 6 of these patients received multiple lines of subsequent treatment. The most common subsequent therapies after progression on nivolumab included: cabozantinib (8 patients), axitinib (6 patients), lenvatinib/everolimus (2 patients) and everolimus (2 patients).

### Safety and treatment-related adverse events

Nivolumab monotherapy was overall well tolerated in this patient cohort and the majority of patients did not miss any intended treatment doses. Nivolumab treatment had to be postponed or delayed at least once for 34% (14/41) of patients in this cohort. Treatment-related adverse events (TRAEs) of any grade were recorded in 37% (15/41) of patients while TRAEs leading to hospitalization occurred in 12% (5/41). There were no treatment-related deaths directly attributable to nivolumab. The most common treatment-related adverse events (noted in > 5% of treated patients) included fatigue/malaise (12%, 5 patients), fever (10%, 4 patients), rash/skin toxicity (10%, 4 patients), and hypothyroidism (7%, 3 patients). Among the more severe TRAEs, one patient had respiratory failure requiring intubation that was potentially related to nivolumab and one developed third-degree heart block. Two patients had Grade 4 febrile reactions necessitating hospitalization. The full list of TRAEs in this patient cohort is shown in Table 3. Among 31 patients in this study who stopped taking nivolumab at the time of analysis, 25 (81%) had discontinuation due to disease progression and 6 patients (19%) had discontinuation due to treatment intolerance.

**Table 3:** Treatment-related adverse events in patients treated with nivolumab

Event	Any grade	Grade 3 or 4
	Number of patients (%)	
Fatigue / Malaise	5 (12%)	1 (2%)
Fever	4 (10%)	3 (7%)
Rash / Skin Toxicity	4 (10%)	2 (5%)
Hypothyroidism	3 (7%)	0
Diarrhea	2 (5%)	1 (2%)
Arthralgia	2 (5%)	0
Myalgia / Myositis	2 (5%)	0
Adrenal Insufficiency	2 (5%)	0
Peripheral Edema	2 (5%)	0
Heart Block (3rd degree)	1 (2%)	1 (2%)
Respiratory Failure	1 (2%)	1 (2%)
Headache	1 (2%)	1 (2%)
Aphasia	1 (2%)	0
Infusion Reaction	1 (2%)	0
Diabetes	1 (2%)	0
Uveitis	1 (2%)	0
Hypertension	1 (2%)	0
Hypophysitis	1 (2%)	0
Cough	1 (2%)	0
Lymphadenopathy	1 (2%)	0
Back Pain	1 (2%)	0

## Discussion

The clinical efficacy of nivolumab monotherapy is established in metastatic RCC with a clear cell histologic component, but has not been reported in patients with non-clear cell histology. In this multicenter retrospective analysis, nivolumab demonstrated an objective response rate of 20% in a heterogeneous population of patients with non-clear cell mRCC and was well tolerated. In non-ccRCC patients, another retrospective analysis has shown potential efficacy of a heterogeneous array of immune checkpoint inhibitors (ICIs) and ICI combinations [17]. That patient series, although suggesting clinical efficacy of ICIs in non-ccRCC, included patients treated with many different immunotherapy agents and combinations, including combinations with anti-VEGF(R) and anti-CTLA4 therapy. The analysis presented here is the first to specifically demonstrate the clinical efficacy of nivolumab monotherapy in patients with non-ccRCC.

Despite the apparent differences in biology and underlying molecular mechanisms between clear cell and non-clear cell tumors, the mechanism of action of nivolumab to activate T cells through inhibition of the PD-1/PDL1 interaction, generates a hypothesis that the clinical activity of nivolumab may not be restricted to clear cell histology. In a prior series of 101 non-ccRCC pathologic specimens, positive PDL1 expression was noted in tumor-infiltrating mononuclear cells (TIMCs) in over half (56%) of the patients, and varied by histology from 36% in chromophobe RCC to 100% in collecting duct carcinoma. PDL1 positivity of tumor cells was also noted in about 10% of biopsies and PDL1 positivity in both tumor cells and TIMCs was associated with worse clinical outcomes [18]. Another study demonstrated higher expression of PDL1 in RCCs with sarcomatoid differentiation [19]. Notably, in patients with ccRCC, the CheckMate 025 trial demonstrated an overall survival benefit of nivolumab over everolimus regardless of PDL1 expression status. PDL1 expression data was not available for the non-clear cell RCC patients in this analysis and thus the association of PDL1 expression and response to nivolumab in non-ccRCC requires further investigation.

The patient population included in this analysis is representative of the patients with non-ccRCC seen in clinical oncology practice. The majority of patients had either papillary, unclassified, chromophobe, or collecting duct histology, consistent with the reported epidemiology of non-ccRCC [2, 3]. Although unclassified histology was the second most common histology in this cohort of non-clear cell patients, there was no evidence from available pathology reports to indicate that this was due to inadequate quality of tissue samples used for pathologic diagnosis or due to the use of less stringent diagnostic criteria. Compared to the CheckMate 025 trial population, the patient population with non-ccRCC presented here was more diverse including more African-American and Hispanic patients. The non-clear cell population was also more heavily pretreated with 10% of patients having had 3 or more prior lines of treatment and all but 3 patients (7.5%) having had prior systemic treatment.

The objective response rate (ORR) observed in this retrospective review at 20% was consistent with the ORR reported in the CheckMate 025 trial. However, a higher percentage of patients with primary PD was noted in the non-ccRCC population at 51%. The response rate was highest for patients with unclassified histology, but responses were seen in three out of four most common non-clear cell histologies (papillary, unclassified, and collecting duct). For chromophobe histology, although no partial responses were observed, three out of four patients had stable disease as best response and two of the patients had a net decrease in the size of tumor lesions. Despite a comparatively short median duration of treatment of 3.0 months, the median time to best response among responders was 5.1 months. PFS was also modest at 3.5 months, consistent with prior trials of nivolumab in RCC. The available data suggest the potential for durable responses to nivolumab in non-ccRCC patients. Among seven patients with documented response to nivolumab, only two had progression during the follow-up period. Interestingly, two patients had their first documented radiographic response at least 4 weeks following discontinuation of nivolumab treatment. Another three patients were treated beyond radiographic progression for a duration of 2–4 months until clinical progression. In conjunction with other retrospective data supporting the efficacy of ICI treatments in non-ccRCC, the results of this analysis additionally support clinical trials that assess the efficacy of ICI monotherapy or ICI combinations for patients with non-clear cell RCC. One such trial assessing the efficacy of another anti-PD-1 agent, pembrolizumab, in both ccRCC and non-ccRCC is currently accruing patients [20].

It is more challenging to assess safety outcomes due to the retrospective nature of this analysis as it is difficult to assign causality as well as grade of the adverse events based on a retrospective chart review. Despite this limitation, nivolumab monotherapy was overall well tolerated in this population of patients with non-ccRCC. The reported TRAEs were consistent with what has previously been reported in patients treated with immunotherapy agents and with nivolumab in particular. The majority of patients did not have dose delays on nivolumab treatment and only a minority had nivolumab discontinued due to adverse events. Importantly, no patient deaths were directly attributable to nivolumab.

This study had a number of other limitations, chief among them being the small sample size and retrospective nature of this analysis with the potential to introduce multiple confounders. Non-ccRCC are rare tumors, thus limiting our ability to include more patients within the specific inclusion criteria of this retrospective analysis. Despite this limitation, by including patients from multiple centers across the United States, a diverse and heterogeneous patient cohort was investigated to address this important clinical question. The lack of a central pathology review was another limitation of this analysis which is frequently encountered in similar retrospective studies. However, all tissue samples used to establish histological diagnosis were reviewed by experienced GU pathologists at high-volume tertiary academic centers that participated in this retrospective analysis. Moreover, in order to establish the histological diagnosis, stringent pathology reviews were applied to high-quality tissue samples. The majority of tissue samples came from nephrectomy specimens and most of the remaining samples from renal biopsies, with only three patients diagnosed with a non-clear cell histology based on biopsies of metastatic lesions. Due to the limited time since the approval of nivolumab for mRCC, the median follow-up of patients in this study was shorter than was necessary to produce data on median duration of treatment response and median OS for patients with non-ccRCC treated with nivolumab. Finally, not all non-clear cell histologies were captured in this series (such as medullary RCC), although the most common non-clear cell subtypes were represented.

## Conclusions

Nivolumab monotherapy demonstrated anti-tumor activity in a population of patients with metastatic non-clear cell renal cell carcinoma. In the absence of available prospective data, this analysis lends support to the use of nivolumab for treatment-refractory patients with metastatic non-ccRCC.

## Abbreviations

ccRCC: clear cell renal cell carcinoma
ICI: Immune checkpoint inhibitors
IMDC: International Metastatic Renal Cell Carcinoma Database Consortium
mRCC: metastatic renal cell carcinoma
MTSCC: Mucinous tubular and spindle cell carcinoma
non-ccRCC: non-clear cell renal cell carcinoma
ORR: Objective response rate
OS: Overall survival
PD: Progressive disease
PFS: Progression free survival
RECIST: Response Evaluation Criteria in Solid Tumors
SD: Stable disease
TIMCs: Tumor-infiltrating mononuclear cells
TRAEs: Treatment-related adverse events
